# Association between RDW-to-albumin ratio and mortality in HFpEF: a retrospective study based on MIMIC-IV and external validation

**DOI:** 10.3389/fnut.2025.1653136

**Published:** 2026-01-16

**Authors:** Zhen Wang, Ting-ting Fan, Meng-li Li, Nin-jun Zhu, Yan-mei Zhang

**Affiliations:** Department of Cardiology, The Second Affiliated Hospital of Anhui Medical University, Hefei, Anhui, China

**Keywords:** heart failure with preserved ejection fraction, inflammation, intensive care, mortality, prognostic biomarker, red-cell-distribution-width-to-albumin ratio

## Abstract

**Objective:**

Heart failure with preserved ejection fraction (HFpEF) in the intensive care unit (ICU) has high mortality, yet reliable bedside prognostic markers remain limited. The red cell distribution width-to-albumin ratio (RDW/Alb), reflecting inflammation and nutrition, has not been validated in this setting.

**Methods:**

This retrospective cohort study queried the MIMIC-IV (v2.2) database for adults (≥18 years) with first ICU admission and HFpEF (left ventricular ejection fraction ≥50% by ICD coding or echocardiographic narrative). RDW and serum albumin within 24 h of ICU entry were used to calculate RDW/Alb, analyzed as tertiles (T1 ≤ 4.08; T2 4.08–5.13; T3 > 5.14). The primary endpoint was all-cause mortality at 30, 90, and 365 days. Kaplan–Meier curves, multivariable Cox regression, restricted cubic splines (RCS), and subgroup analyses were conducted. Prognostic discrimination of RDW/Alb was compared with the triglyceride-glucose (TyG) index in a biomarker-complete subset. Findings were externally validated in 429 HFpEF patients from general wards at our hospital.

**Results:**

Among 3,436 ICU-HFpEF patients, 659 (19.2%), 907 (26.4%), and 2,997 (87.3%) deaths occurred at 30, 90, and 365 days, respectively. Mortality rose stepwise across tertiles (30-day: 8.0% vs 16.2% vs 33.3%; log-rank < 0.001). In fully adjusted models, each unit increase in RDW/Alb was associated with 12% higher hazard for 30-day (HR 1.12, 95% CI 1.10–1.15) and 90-day mortality (HR 1.12), and a 10% increase for 1-year mortality (HR 1.10, 95% CI 1.07–1.12). Compared with T1, T3 patients had HRs of 3.13, 3.02, and 1.37 for 30-, 90-, and 365-day mortality (all *p* < 0.001). RCS revealed a nonlinear risk surge above an RDW/Alb of 4.56. The association remained across subgroups and was stronger in females, non-diabetics, and non-statin users (interaction < 0.01). In 490 patients with glucose and triglyceride data, RDW/Alb outperformed TyG in predicting mortality (AUC 0.67–0.68 vs 0.52–0.54; *p* < 0.01). External validation confirmed RDW/Alb as a predictor of 1-year mortality (HR for T3 vs T1: 2.90; 95% CI: 1.55–5.41; *p* < 0.001).

**Conclusion:**

RDW/Alb is a simple, widely available marker that strongly predicts mortality in ICU patients with HFpEF, outperforming TyG and supporting its role in early risk stratification.

## Introduction

HFpEF is a prevalent and challenging clinical syndrome characterized by diastolic dysfunction and elevated filling pressures, associated with significant morbidity and mortality (1). Despite therapeutic advances, patients with HFpEF continue to experience frequent hospitalizations and high short-term mortality (2). The burden is particularly severe in the ICU setting: in the United States, approximately 10–51% of hospitalized HF patients require ICU care, reflecting a critically ill subgroup with substantially elevated mortality risk (3). In light of the absence of HFpEF-specific therapies that improve survival, accurate risk stratification is essential (4). Identifying novel prognostic factors in critically ill HFpEF patients may guide clinical decision-making and optimize resource allocation.

In recent years, increasing attention has been given to metabolic and inflammatory biomarkers for improving risk prediction in HFpEF and other acute cardiac conditions. For example, the triglyceride-glucose (TyG) index—a surrogate for insulin resistance—has emerged as a promising predictor of adverse outcomes. A high TyG index is associated with increased long-term mortality and rehospitalization in HFpEF, underscoring the role of metabolic dysfunction in its prognosis (5). Similarly, the stress hyperglycemia ratio (SHR), which reflects acute glycemic derangement relative to baseline glucose control, has been identified as a prognostic marker in ICU settings. However, its U-shaped association with mortality suggests that both hypoglycemia and hyperglycemia may be detrimental (6). Systemic inflammation is another important contributor to HFpEF progression. Elevated C-reactive protein (CRP) levels strongly predict worse outcomes. For instance, in the TOPCAT trial, patients with high-sensitivity CRP ≥2 mg/L had more than twice the risk of cardiovascular death or heart failure hospitalization (7). Moreover, hematologic indices such as the neutrophil-to-lymphocyte ratio (NLR) and platelet-to-lymphocyte ratio (PLR)—reflecting systemic inflammation and physiologic stress—have been linked to poor outcomes in both chronic and acute HF. In a multicenter cohort of decompensated HFpEF, patients with elevated NLR and PLR had a nearly threefold higher risk of post-discharge cardiac death (8).

Despite their promise, these biomarkers—such as TyG and SHR—have not been systematically evaluated in critically ill HFpEF patients. Their prognostic utility in this high-risk subgroup remains uncertain. Additionally, several laboratory components required to calculate these indices, such as serum triglycerides, are not routinely tested in HFpEF patients, which limits their practicality in the ICU setting. Similarly, although inflammatory markers like CRP, NLR, and PLR are widely available, they lack disease specificity and can be confounded by infection, comorbidities, or other acute stressors unrelated to heart failure severity.

A potentially valuable alternative is the red cell distribution width to albumin ratio (RDW/Alb, also termed RAR), a simple composite biomarker that integrates two routinely available parameters. RDW reflects anisocytosis and systemic inflammation, while serum albumin serves as a marker of nutritional status and systemic inflammatory burden (9). Both components are pathophysiologically relevant in HFpEF: chronic low-grade inflammation and oxidative stress can impair erythropoiesis and elevate RDW (10), whereas hypoalbuminemia indicates poor nutritional reserve and ongoing inflammatory processes (11). Individually, elevated RDW has been associated with increased all-cause mortality in heart failure (12, 13), and low serum albumin is a well-known predictor of adverse outcomes in both acute and chronic disease states (14). The RDW/Alb ratio has shown strong prognostic performance in the general population and among ICU patients with coronary artery disease and diabetes mellitus, correlating with higher all-cause mortality (9, 15).

Notably, while RDW/Alb has demonstrated prognostic value in various clinical settings, it has not been specifically evaluated in critically ill patients with HFpEF. Prior studies have primarily focused on patients with acute illnesses or heterogeneous heart failure cohorts (16). To our knowledge, no study has directly examined the utility of RDW/Alb in the ICU setting for HFpEF—a population characterized by both chronic comorbidities and acute hemodynamic compromise. We hypothesize that RDW/Alb, by capturing the interplay between inflammation and nutritional status, may serve as a reliable prognostic biomarker in ICU patients with HFpEF. Therefore, the present study aimed to investigate whether RDW/Alb independently predicts 30-, 90-, and 365-day all-cause mortality among critically ill patients with HFpEF. Additionally, we sought to externally validate our findings in an independent cohort of non-ICU HFpEF patients, assessing the robustness and generalizability of RDW/Alb as a prognostic biomarker across different clinical settings.

## Materials and methods

### Data source

This retrospective cohort study utilized two independent data sources. The primary cohort was extracted from the publicly available MIMIC-IV (v2.2) database, which contains de-identified health records of patients admitted to intensive care units at the Beth Israel Deaconess Medical Center between 2008 and 2019. The database includes comprehensive clinical data such as demographics, laboratory tests, diagnoses, procedures, and survival outcomes. Access to the database was granted after completion of the required training and certification (Record ID: [69335907]).

For external validation, we retrospectively collected data from the Second Affiliated Hospital of Anhui Medical University, encompassing adult patients hospitalized in general wards with HFpEF between January 2018 and March 2024. Patient data—including baseline characteristics, laboratory tests, comorbidities, medications, and outcomes—were retrieved from the hospital's electronic medical record system. Vital status at 365 days was determined by telephone follow-up or electronic medical records. This cohort allowed us to validate the predictive value of RDW/Alb outside the ICU setting and assess its generalizability to general-ward HFpEF patients. This retrospective study was approved by the Ethics Committee of the Second Affiliated Hospital of Anhui Medical University (Approval No. [SL-YX2025-013]).

### Study population – primary cohort (MIMIC-IV)

We identified adult patients (aged ≥18 years) with heart failure with preserved ejection fraction (HFpEF) during their first ICU stay. To improve the accuracy of HFpEF identification, we used a two-step approach: First, patients were screened using the International Classification of Diseases (ICD-9/10) codes for HFpEF and unspecified heart failure (see Supplementary Table 1). Second, for those without clearly coded HF subtypes, we extracted left ventricular ejection fraction (LVEF) values from unstructured clinical narratives in the MIMIC-IV note module, including discharge summaries and echocardiogram interpretations. Patients with a documented LVEF ≥50% were classified as HFpEF. Finally, patients identified by either method were combined and duplicates removed. This hybrid strategy was adopted because HFpEF is frequently under-coded in routine clinical documentation, and relying solely on ICD codes may result in under-ascertainment. To evaluate the prognostic value of the RDW/Alb ratio, we included only those patients with complete data on red cell distribution width (RDW) and serum albumin within 24 hours of ICU admission. For comparison with the triglyceride-glucose (TyG) index, a subset of patients with additional glucose and triglyceride measurements available within the same time frame was used.

### Study population – external validation cohort

For external validation, we retrospectively enrolled patients diagnosed with HFpEF at the Second Affiliated Hospital of Anhui Medical University. Exclusion criteria included: (1) age < 18 years; (2) missing data on RDW or serum albumin within 24 hours of admission; and (3) loss to follow-up within 30 days post-discharge. Only the first eligible admission was analyzed for patients with multiple hospitalizations.

### Exposure variables

The primary exposure variable was the red cell distribution width-to-albumin ratio (RDW/Alb), calculated by dividing RDW (%) by serum albumin concentration (g/dL). For descriptive and survival analyses, RDW/Alb values were categorized into tertiles based on the distribution within each cohort.

In the primary cohort (MIMIC-IV), the triglyceride-glucose (TyG) index was also calculated using the formula:


$$\begin{array}{l} \text{TyG}=\text{ln}\left[\text{fastingtriglycerides}\left(\text{mg}/\text{dL}\right)\times glucose\;\left(mg/dL\right)/2\right] \end{array}$$

Additional variables extracted included age, sex, body mass index (BMI), vital signs, comorbidities (e.g., diabetes, hypertension, chronic kidney disease), and laboratory indices (e.g., white blood cell count, hemoglobin, creatinine). Severity scores such as OASIS, SOFA, and SAPS II were obtained in the MIMIC-IV cohort to adjust for baseline illness severity.

In the external validation cohort, the same RDW/Alb calculation and covariates were used wherever available; however, illness severity scores (e.g., SOFA, OASIS) and TyG index were not routinely collected and therefore were not analyzed in that cohort.

### Outcomes

The primary outcome was all-cause mortality at 30-, 90-, and 365-days following ICU admission in the MIMIC-IV cohort. Time-to-event was defined from ICU admission to the date of death or the end of follow-up, whichever occurred first. In the external validation cohort, only 365-day all-cause mortality was assessed, with time-to-event calculated from hospital admission to death or the end of 1-year follow-up.

### Statistical analysis

Baseline characteristics were summarized across tertiles of the RDW/Alb ratio. Continuous variables were reported as means ± standard deviations or medians with interquartile ranges, and categorical variables as frequencies with percentages. Group comparisons were performed using Student's t-test, Mann–Whitney U test, or chi-square test, as appropriate. In the MIMIC-IV cohort, the prognostic performance of RDW/Alb and TyG was assessed using Kaplan–Meier survival curves and log-rank tests at 30, 90, and 365 days. Cox proportional hazards models were constructed to estimate hazard ratios (HRs) and 95% confidence intervals (CIs), adjusting for potential confounders in stepwise models. Restricted cubic spline (RCS) analysis was used to explore nonlinear associations between RDW/Alb and mortality. Receiver operating characteristic (ROC) curves were plotted to compare the discriminative ability of RDW/Alb and TyG, and area under the curve (AUC) values were compared using DeLong's test. For the external validation cohort, only 365-day all-cause mortality was analyzed. Kaplan–Meier curves and Cox models were used to assess the association between RDW/Alb and 1-year mortality. All analyses were performed using R software (version 4.5.0), and a two-sided *P*-value < 0.05 was considered statistically significant.

## Results

### Baseline characteristics of study subjects

**Figure 1:**
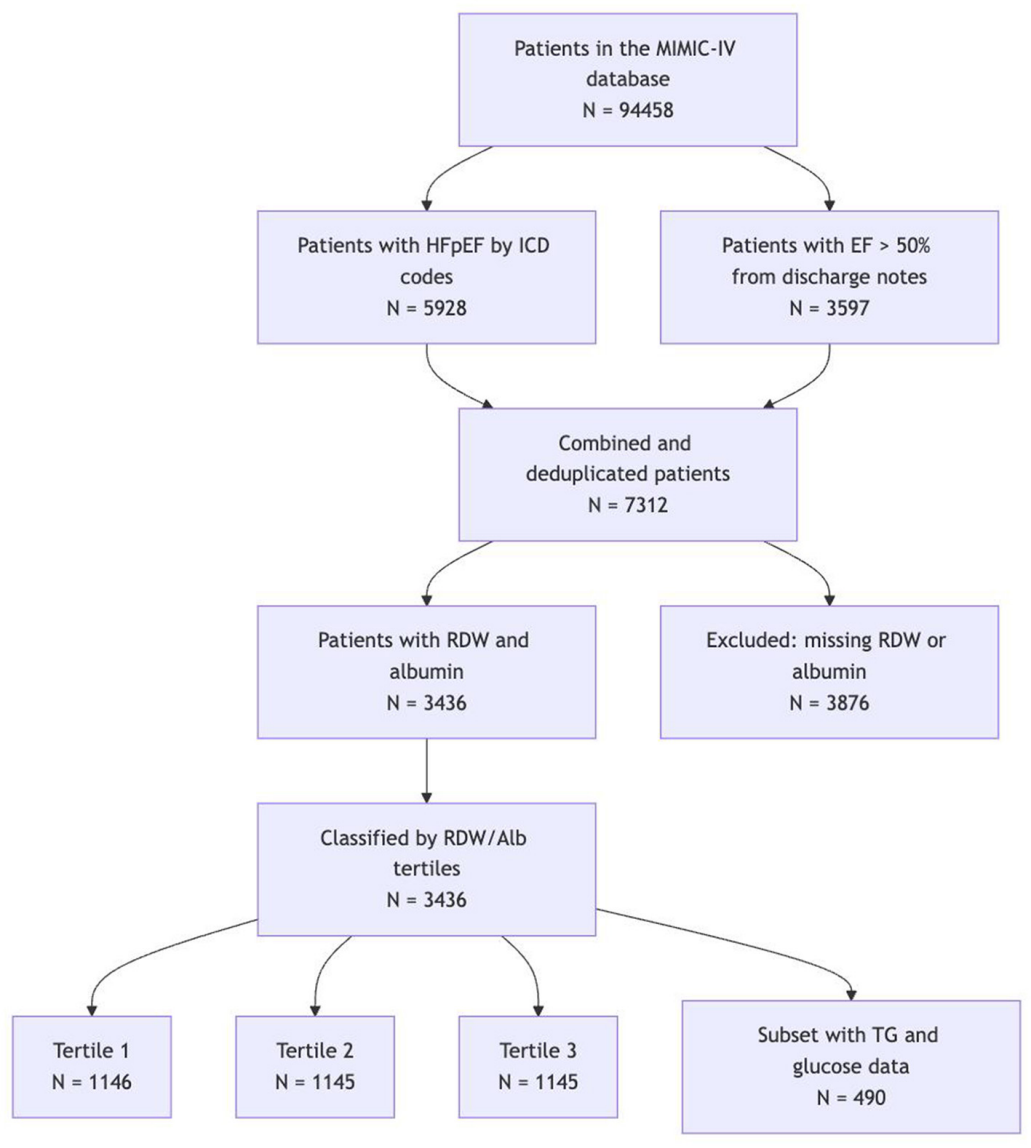
Flowchart of the study cohort.

A total of 3436 ICU patients with HFpEF were included in the final analysis (see Figure 1). Patients were stratified into tertiles based on the RDW/Albumin ratio: T1 ( ≤ 4.08), T2 (4.08–5.14), and T3 (>5.14), with 1146, 1145, and 1145 patients in each group, respectively. Patients in the highest RDW/Alb tertile (T3) were more likely to be male and had slightly higher BMI compared to those in the lowest tertile (T1). Although age was comparable across tertiles, racial distribution differed significantly, with a decreasing proportion of White patients from T1 to T3. From T1 to T3, there was an increasing trend in heart rate, respiratory rate, creatinine, urea nitrogen, white blood cell count, AST, total bilirubin, and OASIS scores, indicating progressively worse clinical status. Conversely, hemoglobin, bicarbonate, sodium, and MAP showed a downward trend. The baseline characteristics are summarized in Table 1 and Supplementary Table 3.

**Table 1:** Baseline characteristics of participants classified by RDW/Alb tertiles.

**Characteristics**	**T1 (*n* = 1,146)**	**T2 (*n* = 1,145)**	**T3 (*n* = 1,145)**	***P*-value**
Age, years	74.0 (17.8)	74.0 (19.0)	74.0 (19.0)	0.409
Female, *n* (%)	599 (52.3%)	536 (46.8%)	537 (46.9%)	0.011
Race: White, *n* (%)	856 (74.7%)	793 (69.3%)	772 (67.4%)	< 0.001
Weight, kg	80.0 (28.0)	79.2 (32.5)	81.0 (32.2)	0.471
BMI, kg/m^2^	28.4 (8.9)	28.5 (11.0)	29.4 (11.8)	0.001
Heart rate, bpm	80.0 (20.0)	85.0 (26.0)	88.0 (29.0)	< 0.001
MAP, mmHg	77.0 (19.0)	76.0 (20.0)	76.0 (23.0)	0.111
RR, bpm	17.0 (8.0)	19.0 (8.0)	20.0 (8.0)	< 0.001
SpO_2_,%	98.0 (5.0)	98.0 (5.0)	97.0 (6.0)	< 0.001
Bicarbonate, mmol/L	25.0 (5.0)	24.0 (7.0)	23.0 (7.0)	< 0.001
Chloride, mmol/L	102.0 (6.0)	101.0 (8.0)	102.0 (9.0)	0.006
Creatinine, mg/dL	1.1 (0.6)	1.3 (1.1)	1.4 (1.4)	< 0.001
Glucose, mmol/L	123.0 (53.0)	126.0 (66.0)	121.0 (65.0)	0.074
Hemoglobin, g/L	12.0 (2.5)	10.5 (2.8)	9.4 (2.8)	< 0.001
Magnesium, mmol/L	2.0 (0.4)	2.0 (0.4)	2.0 (0.4)	0.794
Platelet count, 10^9^/L	198.0 (90.0)	210.0 (122.0)	195.0 (144.0)	0.002
Potassium, mmol/L	4.2 (0.7)	4.2 (0.9)	4.2 (0.9)	0.062
Sodium, mmol/L	139.0 (4.0)	138.0 (6.0)	138.0 (6.0)	< 0.001
Urea nitrogen	23.0 (17.0)	29.0 (26.0)	32.0 (32.0)	< 0.001
WBC, 10^9^/L	8.3 (4.6)	9.7 (6.3)	10.6 (8.8)	< 0.001
ALT	21.0 (18.0)	22.0 (25.0)	22.0 (29.0)	0.828
AST	26.0 (20.0)	30.0 (34.0)	33.0 (43.0)	< 0.001
Bilirubin total	0.5 (0.4)	0.6 (0.6)	0.7 (0.9)	< 0.001
SOFA score	8.0 (8.0)	6.0 (9.0)	7.0 (9.0)	< 0.001
SAPS II score	67.0 (26.0)	61.0 (27.0)	63.0 (26.0)	< 0.001
OASIS	37.0 (9.0)	38.0 (10.0)	40.0 (11.0)	< 0.001
RRT use, *n* (%)	31 (2.7%)	38 (3.3%)	90 (7.9%)	< 0.001
Vasoactive drug, *n* (%)	529 (46.2%)	452 (39.5%)	509 (44.5%)	0.004
Ventilator use, *n* (%)	675 (58.9%)	526 (45.9%)	578 (50.5%)	< 0.001
Smoking, *n* (%)	225 (19.6%)	237 (20.7%)	198 (17.3%)	0.106
Used aspirin	921 (80.4%)	764 (66.7%)	585 (51.1%)	< 0.001
Used clopidogrel	299 (26.1%)	203 (17.7%)	120 (10.5%)	< 0.001
Used dabigatran	7 (0.6%)	11 (1.0%)	8 (0.7%)	0.603
Used rivaroxaban	42 (3.7%)	31 (2.7%)	26 (2.3%)	0.125
Used warfarin	394 (34.4%)	353 (30.8%)	276 (24.1%)	< 0.001
Used heparin	1,051 (91.7%)	1,051 (91.8%)	1,070 (93.4%)	0.211
Used beta blocker	973 (84.9%)	913 (79.7%)	803 (70.1%)	< 0.001
Used ACEI or ARB	544 (47.5%)	442 (38.6%)	268 (23.4%)	< 0.001
Used ARNI	5 (0.4%)	7 (0.6%)	1 (0.1%)	0.087
Used CCB	490 (42.8%)	479 (41.8%)	359 (31.4%)	< 0.001
Used digitalis	103 (9.0%)	98 (8.6%)	115 (10.0%)	0.449
Used diuretic	1,017 (88.7%)	1,008 (88.0%)	960 (83.8%)	< 0.001
Used amiodarone	278 (24.3%)	214 (18.7%)	205 (17.9%)	< 0.001
Used insulin	892 (77.8%)	769 (67.2%)	755 (65.9%)	< 0.001
Used statin	817 (71.3%)	757 (66.1%)	616 (53.8%)	< 0.001
Used SGLT2-i	4 (0.3%)	2 (0.2%)	1 (0.1%)	0.519
Used metformin	78 (6.8%)	39 (3.4%)	23 (2.0%)	< 0.001
Used MRA	81 (7.1%)	100 (8.7%)	126 (11.0%)	0.004
Diabetes, *n* (%)	431 (37.6%)	519 (45.3%)	505 (44.1%)	< 0.001
Hypertension, *n* (%)	344 (30.0%)	274 (23.9%)	201 (17.6%)	< 0.001
Myocardial infarction, *n* (%)	203 (17.7%)	220 (19.2%)	165 (14.4%)	0.008
Coronary artery disease, *n* (%)	589 (51.4%)	487 (42.5%)	417 (36.4%)	< 0.001
Cancer, *n* (%)	90 (7.9%)	170 (14.8%)	247 (21.6%)	< 0.001
Atrial fibrillation, *n* (%)	329 (28.7%)	346 (30.2%)	365 (31.9%)	0.256
Acute kidney injury, *n* (%)	439 (38.3%)	618 (54.0%)	704 (61.5%)	< 0.001
Chronic kidney disease, *n* (%)	393 (34.3%)	510 (44.5%)	526 (45.9%)	< 0.001
COPD, *n* (%)	107 (9.3%)	195 (17.0%)	177 (15.5%)	< 0.001
Sepsis, *n* (%)	56 (4.9%)	128 (11.2%)	261 (22.8%)	< 0.001
Hyperlipidemia, *n* (%)	697 (60.8%)	598 (52.2%)	512 (44.7%)	< 0.001
Hyperthyroidism, *n* (%)	12 (1.0%)	9 (0.8%)	11 (1.0%)	0.803
Cerebrovascular disease, *n* (%)	86 (7.5%)	72 (6.3%)	76 (6.6%)	0.493
Paraplegia, *n* (%)	6 (0.5%)	2 (0.2%)	14 (1.2%)	0.008
Length of ICU stay, day	2.3 (2.9)	2.4 (3.0)	3.1 (4.8)	< 0.001
Length of hospital stay, day	9.0 (7.6)	10.6 (10.4)	12.6 (14.9)	< 0.001
Ventilation-free days at 28 days, day	25.7 (2.9)	25.6 (3.0)	24.9 (4.8)	< 0.001
Vasopressor-free days at 28 days, day	25.7 (2.9)	25.6 (3.0)	24.9 (4.8)	< 0.001
ICU-free days at 28 days, day	25.7 (2.9)	25.6 (3.0)	24.9 (4.8)	< 0.001

Continuous variables are presented as mean ± standard deviation or median (interquartile range), as appropriate based on the distribution of each variable; categorical variables are shown as number (%).

BMI, body mass index; RRT, renal replacement therapy; OASIS, Oxford acute severity of illness score; SAPS II, Simplified Acute Physiology Score II; SOFA, Sequential Organ Failure Assessment; MAP, mean arterial pressure; RR, respiratory rate; SpO_2_, pulse oximetry-derived oxygen saturation; WBC, white blood cell; ICU, intensive care unit; ALT, alanine aminotransferase; AST, aspartate aminotransferase; ACEI, angiotensin-converting enzyme inhibitor; ARB, angiotensin receptor blocker; ARNI, angiotensin receptor–neprilysin inhibitor; CCB, calcium channel blocker; SGLT2-i, sodium–glucose cotransporter-2 inhibitor; MRA, mineralocorticoid receptor antagonist; COPD, chronic obstructive pulmonary disease.

In terms of comorbidities, the prevalence of diabetes, cancer, sepsis, acute kidney injury, and chronic kidney disease increased across tertiles, while hypertension and hyperlipidemia showed a decreasing pattern. Use of medications such as insulin, aspirin, clopidogrel, beta-blockers, ACEI/ARB, and statins declined notably from T1 to T3. Meanwhile, the use of renal replacement therapy increased significantly in higher tertiles. Furthermore, patients in T3 had longer ICU and hospital stays and fewer ICU-, ventilator-, and vasopressor-free days within the first 28 days.

### RDW/Alb and mortality

**Figure 2:**
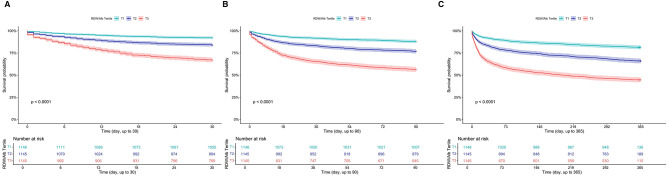
Kaplan–Meier survival analysis curve for all-cause mortality in the overall study population. **(A)** 30-day mortality; **(B)** 90-day mortality; **(C)** 365-day mortality. RDW/Albumin ratio: T1 (≤4.08), T2 (4.08–5.14), and T3 (>5.14).

Of the 3436 patients, 659 (19.2%), 907 (26.4%), and 2997 (87.3%) died within 30, 90, and 365 days of follow-up, respectively. When stratified by RDW/Alb tertiles, mortality showed a progressive increase across groups. In T1, 92 (8.0%), 140 (12.2%), and 1011 (88.2%) patients died at 30, 90, and 365 days, respectively. In T2, the corresponding numbers were 186 (16.2%), 266 (23.2%), and 956 (83.5%). T3 exhibited the highest mortality rates, with 381 (33.3%), 501 (43.8%), and 1030 (90.0%) deaths at each respective time point. Kaplan–Meier survival analysis revealed significant differences in survival among the three groups. Patients in the highest RDW/Alb tertile had markedly worse survival at all follow-up time points. The survival curves demonstrated clear separation across tertiles, with log-rank *P*-values all < 0.001, indicating statistically robust differences (Figure 2).

Using Cox proportional hazards regression, we assessed the association between the RDW/Alb ratio and mortality at 30, 90, and 365 days. When analyzed as a continuous variable, RDW/Alb was significantly associated with increased risk of death at all time points. In fully adjusted Model 3, each unit increase in RDW/Alb was associated with a 12% higher risk of 30-day mortality [HR (95% CI): 1.12 (1.10–1.15), *P* < 0.001], an identical 12% increase for 90-day mortality [HR: 1.12 (1.10–1.15), *P* < 0.001], and a 10% increase for 365-day mortality [HR: 1.10 (1.07–1.12), *P* < 0.001]. When stratified by tertiles, patients in the highest RDW/Alb tertile (T3) had significantly elevated risks of death compared with those in the lowest tertile (T1). For 30-day mortality, the hazard ratio for T3 in Model 3 was 3.13 (2.42–4.03, *P* < 0.001); for 90-day mortality, it was 3.02 (2.45–3.73, *P* < 0.001); and for 365-day mortality, it was 1.37 (1.24–1.52, *P* < 0.001). These results demonstrate a consistent and robust association between higher RDW/Alb levels and increased risk of short- and long-term mortality in ICU patients with HFpEF (for details see Table 2 and Supplementary Table 2).

**Table 2:** Multivariate Cox regression analyses for 30-day, 90-day, and 365-day mortality.

**Variable**	**Model 1 HR (95%CI)**	***P*-value**	**Model 2 HR (95%CI)**	***P*-value**	**Model 3 HR (95%CI)**	***P*-value**
**30-day mortality**						
RDW/Alb (continuous)	1.14 (1.12–1.16)	< 0.001	1.15 (1.12–1.17)	< 0.001	1.12 (1.10–1.15)	< 0.001
T1	1 (Ref)		1 (Ref)		1 (Ref)	
T2	2.13 (1.66–2.73)	< 0.001	1.84 (1.43–2.37)	< 0.001	1.74 (1.34–2.26)	< 0.001
T3	4.82 (3.83–6.05)	< 0.001	3.8 (2.99–4.82)	< 0.001	3.13 (2.42–4.03)	< 0.001
**90-day mortality**						
RDW/Alb (continuous)	1.13 (1.12–1.15)	< 0.001	1.14 (1.12–1.16)	< 0.001	1.12 (1.10–1.15)	< 0.001
T1	1 (Ref)		1 (Ref)		1 (Ref)	
T2	2.04 (1.66–2.50)	< 0.001	1.79 (1.45–2.20)	< 0.001	1.67 (1.35–2.07)	< 0.001
T3	4.44 (3.68–5.36)	< 0.001	3.57 (2.93–4.34)	< 0.001	3.02 (2.45–3.73)	< 0.001
**365-day mortality**						
RDW/Alb (continuous)	1.11 (1.10–1.13)	< 0.001	1.1 (1.09–1.12)	< 0.001	1.1 (1.07–1.12)	< 0.001
T1	1 (Ref)		1 (Ref)		1 (Ref)	
T2	1.03 (0.94–1.13)	0.484	0.99 (0.91–1.09)	0.868	0.99 (0.90–1.09)	0.912
T3	1.54 (1.41–1.68)	< 0.001	1.41 (1.29–1.55)	< 0.001	1.37 (1.24–1.52)	< 0.001

RDW/Alb, Red Cell Distribution Width to Albumin ratio. T1: reference group. RDW/Alb index: T1 (RDW/Alb index ≤ 4.08), T2 (4.08 < RDW/Alb index ≤ 5.14), T3 (RDW/Alb index >5.14). HR, hazard ratio; CI, confidential interval.

Model 1: No adjusted.

Model 2: Adjusted for age, gender, race, weight, diabetes, hypertension, myocardial infarction, coronary artery disease, cancer, atrial fibrillation, chronic kidney disease, COPD, sepsis, hyperlipidemia, cerebrovascular disease.

Model 3: Adjusted for age, gender, race, weight, diabetes, hypertension, myocardial infarction, coronary artery disease, cancer, atrial fibrillation, chronic kidney disease, COPD, sepsis, hyperlipidemia, cerebrovascular disease, RRT, vasoactive drug, ventilator use, heart rate, MAP, smoking, white blood cells, hemoglobin, creatinine, potassium, sodium, used digitalis, used diuretic, used amiodarone, used insulin, used statin, used metformin, used MRA.

**Figure 3:**
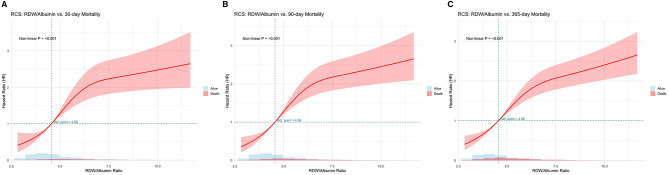
Association between RDW/Alb and all-cause mortality in critically ill patients with HFpEF. **(A)** 30-day mortality; **(B)** 90-day mortality; **(C)** 365-day mortality.

Furthermore, RCS regression analysis revealed a significant nonlinear association between the RDW/Alb ratio and all-cause mortality at 30, 90, and 365 days (*P* for nonlinearity < 0.001 for all). As shown in Figure 3, the risk of mortality began to increase steeply when the RDW/Alb ratio exceeded approximately 4.56. The hazard ratios continued to rise across the full spectrum of RDW/Alb values, with widening confidence intervals at the upper extremes.

### Subgroup analysis

**Figure 4:**
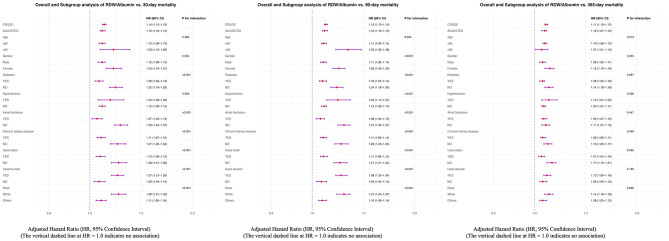
Subgroup analyses of the association between RDW/Alb and all-cause mortality in critically ill patients with HFpEF.

Subgroup analyses were performed to assess whether the association between RDW/Alb ratio and all-cause mortality was consistent across clinically relevant strata, including age, sex, race, hypertension, diabetes, atrial fibrillation, chronic kidney disease, and medication use (statins, diuretics) (Figure 4). The positive association between RDW/Alb and mortality remained consistent across most subgroups, but was notably stronger among females, non-diabetic patients, those not receiving statins, and individuals without atrial fibrillation or chronic kidney disease, with significant interactions observed at 30 and 90 days (*P* for interaction < 0.01). An age-related crossover effect was observed: RDW/Alb showed a stronger predictive value in patients < 65 years at earlier time points, whereas the association became more pronounced in those ≥65 years at 365 days (*P* = 0.012). Despite these variations, RDW/Alb remained significantly associated with mortality across all subgroups, underscoring its robustness as a prognostic marker in critically ill patients with HFpEF.

### Comparison between RDW/Alb and TyG index

**Figure 5:**
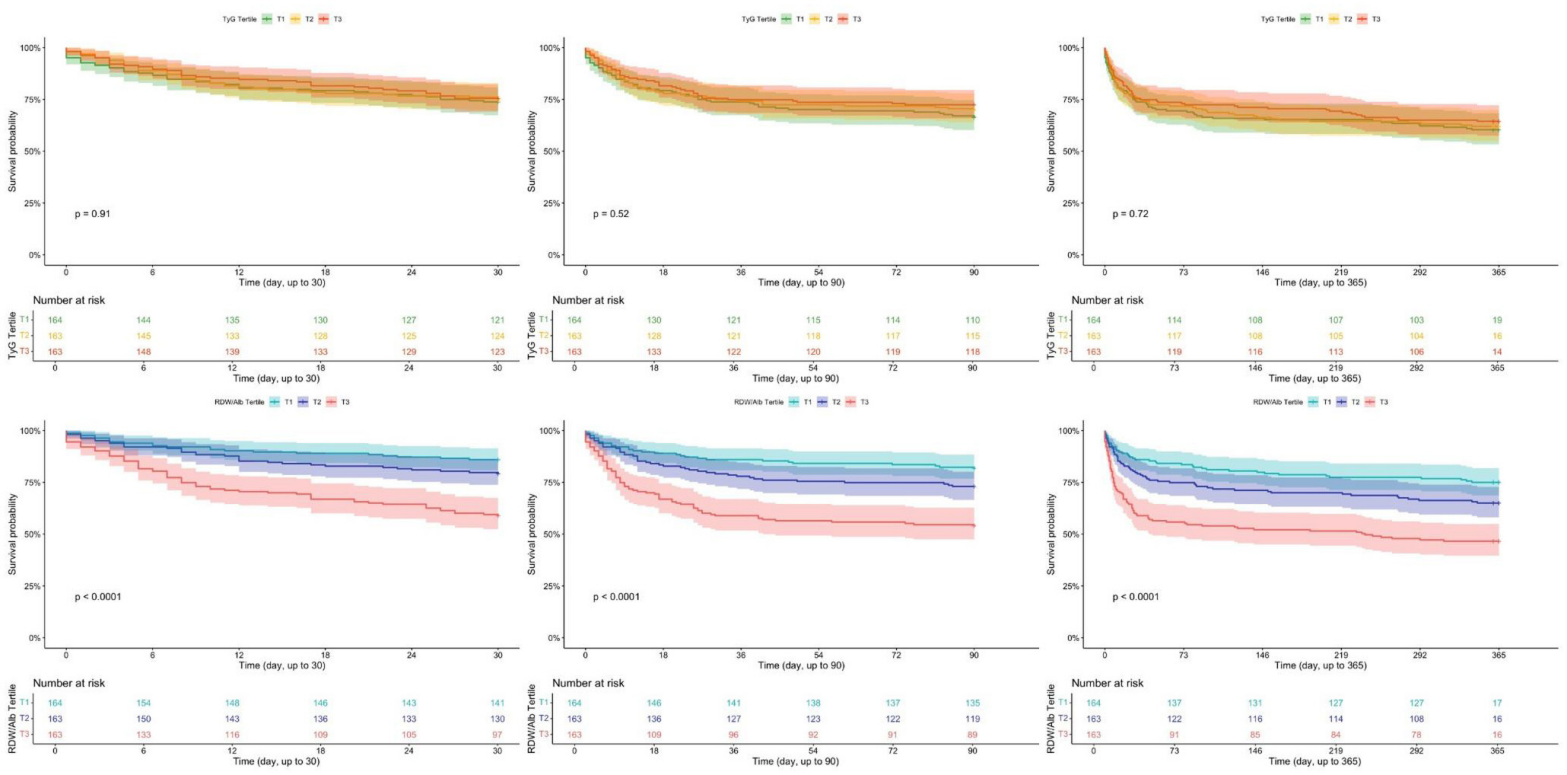
Comparison of prognostic performance between RDW/Albumin and TyG index at 30-, 90-, and 365-days using Kaplan–Meier analyses.

**Figure 6:**
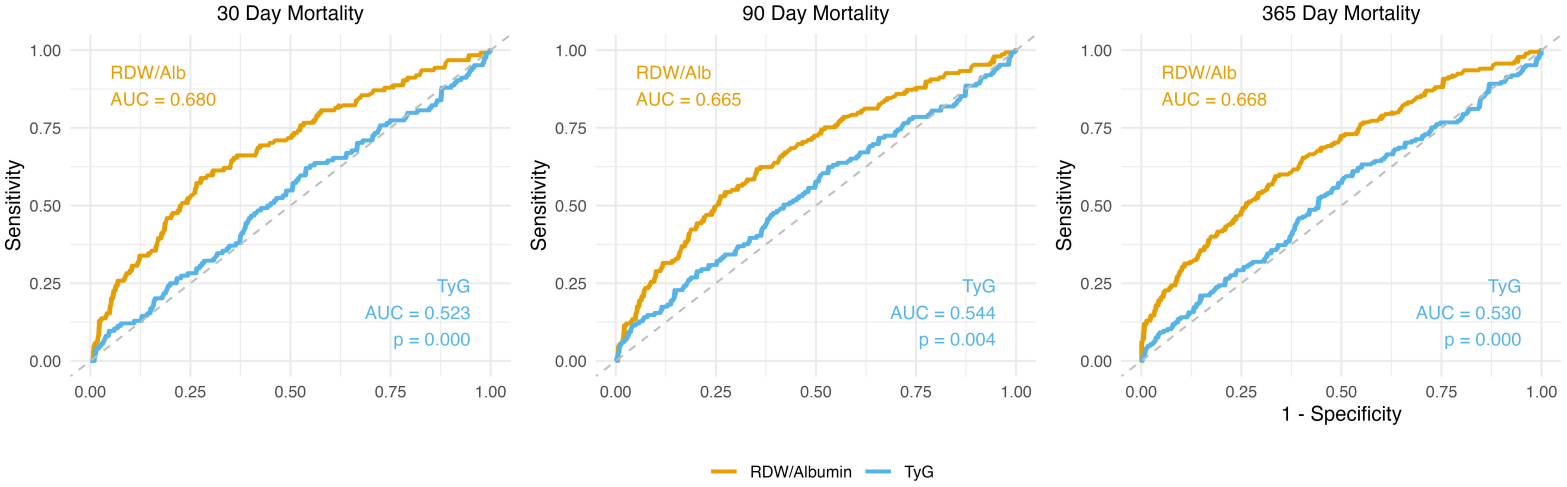
Comparison of prognostic performance between RDW/Albumin and TyG index at 30-, 90-, and 365-days using ROC analyses.

To compare the prognostic performance of RDW/Alb with that of the TyG index, Kaplan–Meier survival analysis and ROC curve analysis were performed in the subset of 490 patients with available data on all relevant biomarkers. As shown in Figure 5, Kaplan–Meier survival curves stratified by TyG tertiles revealed no significant difference in all-cause mortality at 30, 90, or 365 days. The corresponding log-rank *P*-values were 0.91, 0.52, and 0.72, respectively, indicating limited discriminatory capacity of the TyG index in this population. In contrast, ROC curve analysis demonstrated that the RDW/Alb ratio consistently outperformed the TyG index in predicting mortality at all time points. The area under the curve (AUC) for RDW/Alb was 0.680, 0.665, and 0.668 at 30, 90, and 365 days, respectively, compared with 0.523, 0.544, and 0.530 for TyG. All comparisons yielded statistically significant differences (*P* < 0.01), further highlighting the superior prognostic accuracy of RDW/Alb over TyG in critically ill patients with HFpEF (Figure 6).

### External validation in general-ward HFpEF patients

**Figure 7:**
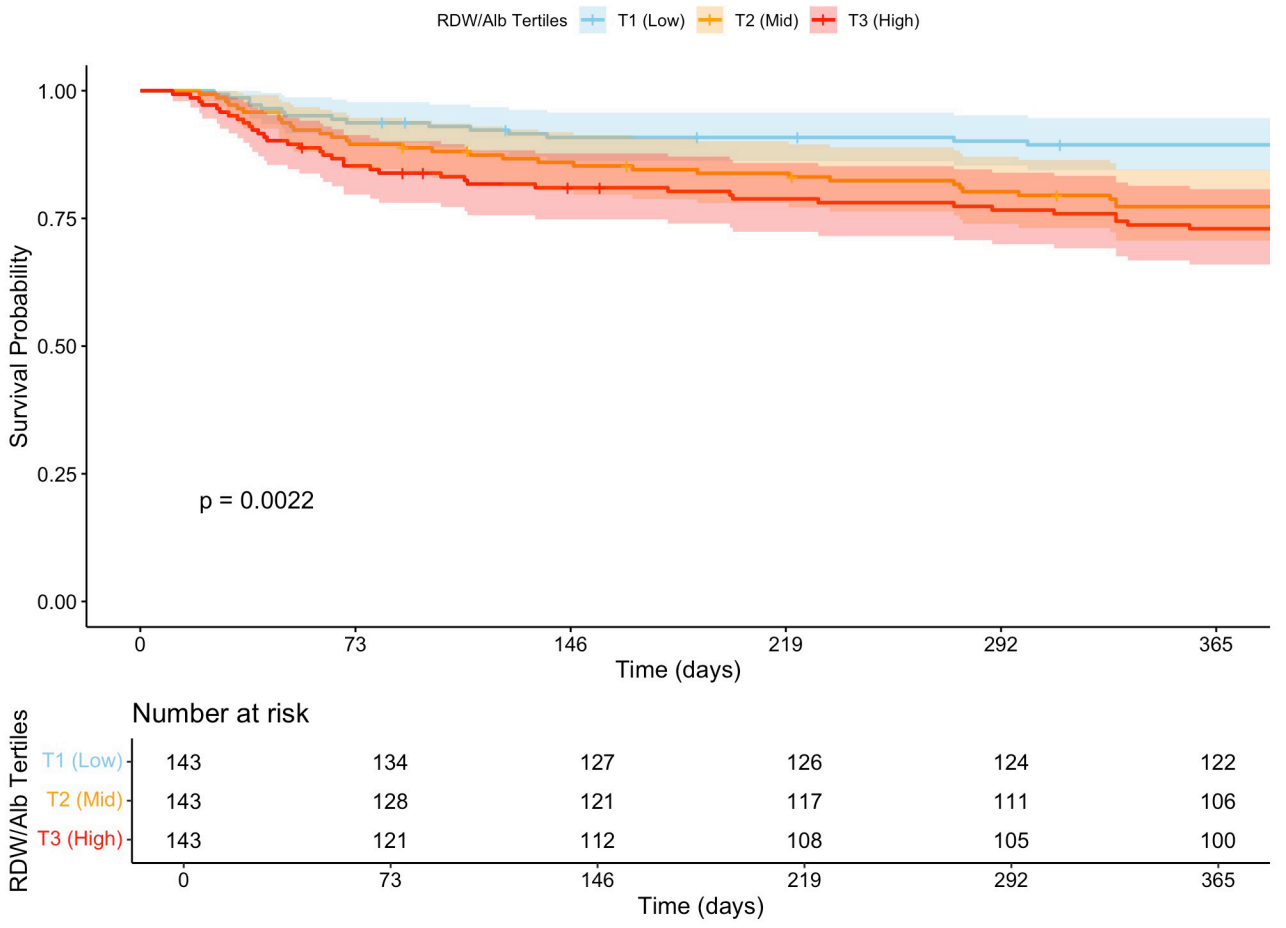
Kaplan–Meier survival curves for 365-day all-cause mortality stratified by RDW/Alb tertiles in the external validation cohort.

To assess the generalizability of our findings, we conducted an external validation in 429 patients hospitalized in general wards with HFpEF. Patients were stratified into tertiles based on RDW/Alb values (T1 < 3.32, T2 (3.32–3.95), and T3 >3.95), which were consistent with the primary cohort. One-year mortality progressively increased across tertiles: 10% in T1, 22% in T2, and 27% in T3 (*P* = 0.001) (for details, see Table 3). Kaplan–Meier survival analysis demonstrated a significant separation of survival curves across RDW/Alb tertiles, with the highest tertile showing markedly reduced survival probability (log-rank *P* = 0.002; Figure 7). In multivariable Cox regression analysis adjusted for age, sex, creatinine, hypertension, and atrial fibrillation, patients in the middle (T2) and highest tertiles (T3) had significantly increased hazards of 365-day mortality compared with the lowest tertile. The adjusted hazard ratio (HR) for T2 was 2.33 (95% CI: 1.25–4.35; *P* = 0.008), and for T3 was 2.90 (95% CI: 1.55–5.41; *P* < 0.001) (Supplementary Figure 1). Subgroup analysis in this external validation cohort revealed that the prognostic value of RDW/Alb remained consistent across multiple clinical strata. The association between higher RDW/Alb and 1-year mortality was particularly pronounced in patients aged ≥65, males, non-diabetics, and those without chronic kidney disease, with hazard ratios ranging from 1.25 to 2.51 across subgroups (see Supplementary Figure 2). These findings reinforce the prognostic value of RDW/Alb and validate its association with long-term mortality in a non-ICU HFpEF population.

**Table 3:** Baseline characteristics of the external validation cohort stratified by RDW/Alb tertiles.

**Characteristics**	**T1 (*n* = 143)**	**T2 (*n* = 143)**	**T3 (*n* = 143)**	***P*-value**
Death (365 days), *n* (%)	15 (10%)	32 (22%)	38 (27%)	0.001
Male, *n* (%)	83 (58%)	70 (49%)	69 (48%)	0.188
RDW, %	13.05 (12.58, 13.50)	13.70 (13.21, 14.20)	15.18 (13.76, 16.53)	< 0.001
Albumin, g/dL	4.27 (4.11, 4.47)	3.84 (3.66, 4.04)	3.30 (2.98, 3.60)	< 0.001
Height, cm	161 (158, 168)	161 (157, 168)	161 (156, 165)	0.075
Weight, kg	68 (62, 75)	68 (61, 75)	68 (60, 72)	0.716
Heart rate, bpm	81 (69, 98)	78 (67, 97)	82 (71, 98)	0.523
WBC, 10^9^/L	6.9 (5.5, 8.6)	6.3 (5.0, 7.6)	7.1 (5.1, 8.8)	0.038
Neutrophil count, 10^9^/L	4.4 (3.6, 6.1)	4.1 (3.2, 5.2)	4.6 (3.4, 6.0)	0.084
Lymphocyte count, 10^9^/L	1.46 (1.06, 2.03)	1.37 (0.99, 1.72)	1.14 (0.84, 1.63)	< 0.001
Hemoglobin, g/L	136 (125, 147)	127 (116, 140)	118 (101, 129)	< 0.001
Platelet count, 10^9^/L	181 (139, 229)	164 (130, 211)	167 (127, 212)	0.051
Triglycerides, mmol/l	1.38 (1.07, 2.24)	1.27 (0.87, 1.69)	1.19 (0.91, 1.60)	< 0.001
Total cholesterol, mmol/l	4.35 (3.63, 4.86)	4.15 (3.41, 4.85)	4.04 (2.98, 4.66)	0.010
HDL-C, mmol/l	1.05 (0.88, 1.26)	1.06 (0.94, 1.31)	1.01 (0.84, 1.18)	0.021
LDL-C, mmol/l	2.71 (2.06, 3.06)	2.60 (2.07, 3.14)	2.36 (1.70, 2.98)	0.029
Sodium, mmol/l	140.6 (138.6, 142.9)	141.0 (139.2, 143.6)	140.7 (137.4, 143.5)	0.253
Chloride, mmol/l	103.7 (100.1, 106.0)	104.8 (102.5, 106.9)	104.0 (100.7, 106.6)	0.007
Potassium, mmol/l	4.17 (4.00, 4.58)	4.18 (3.80, 4.41)	4.09 (3.54, 4.44)	0.003
Calcium, mmol/l	2.24 (2.16, 2.33)	2.22 (2.13, 2.30)	2.13 (2.07, 2.22)	< 0.001
Magnesium, mmol/l	0.84 (0.77, 0.89)	0.84 (0.78, 0.88)	0.84 (0.78, 0.91)	0.642
Uric acid, μmol/L	392 (322, 454)	332 (280, 434)	366 (291, 477)	0.023
Urea nitrogen, mmol/L	6.9 (6.0, 8.1)	7.2 (5.5, 9.0)	8.4 (7.2, 10.6)	< 0.001
Cystatin C, mg/L	1.23 (0.89, 1.49)	1.36 (1.02, 1.80)	1.40 (1.03, 2.02)	< 0.001
Creatinine, μmol/L	81 (64, 106)	87 (64, 111)	95 (81, 120)	< 0.001
NT-proBNP, pg/ml	1,700 (743, 1,700)	1,700 (1,390, 2,522)	1,700 (1,700, 4,324)	< 0.001
Beta blocker*, n* (%)	105 (73%)	102 (71%)	90 (63%)	0.141
ARNI, *n* (%)	89 (62%)	94 (66%)	101 (71%)	0.330
ACEI/AEB, *n* (%)	20 (14%)	30 (21%)	20 (14%)	0.202
MRA, *n* (%)	94 (66%)	101 (71%)	109 (76%)	0.158
Metformin, *n* (%)	42 (29%)	38 (27%)	26 (18%)	0.069
Furosemide, *n* (%)	71 (50%)	91 (64%)	115 (80%)	< 0.001
Antiplatelet, *n* (%)	70 (49%)	50 (35%)	50 (35%)	0.022
Anticoagulation, *n* (%)	40 (28%)	62 (43%)	64 (45%)	0.005
Digoxin, *n* (%)	16 (11%)	18 (13%)	15 (10%)	0.893
Statin, *n* (%)	105 (73%)	94 (66%)	97 (68%)	0.360
Febuxostat, *n* (%)	6 (4.2%)	5 (3.5%)	10 (7.0%)	0.457
Diabetes mellitus, *n* (%)	101 (71%)	83 (58%)	96 (67%)	0.074
Bradyarrhythmia, *n* (%)	10 (7.0%)	10 (7.0%)	11 (7.7%)	>0.999
Valvular heart disease, *n* (%)	20 (14%)	18 (13%)	21 (15%)	0.908
Coronary artery disease, *n* (%)	86 (60%)	75 (52%)	78 (55%)	0.412
Atrial fibrillation, *n* (%)	46 (32%)	69 (48%)	76 (53%)	< 0.001
Malignancy, *n* (%)	1 (0.7%)	8 (5.6%)	11 (7.7%)	0.007
COPD, *n* (%)	15 (10%)	15 (10%)	16 (11%)	>0.999
Hyperlipidemia, *n* (%)	24 (17%)	26 (18%)	15 (10%)	0.158
Hypertension, *n* (%)	100 (70%)	108 (76%)	100 (70%)	0.491
Chronic kidney disease, *n* (%)	12 (8.4%)	16 (11%)	23 (16%)	0.135

## Discussion

In this two-cohort study of patients with HFpEF—critically ill individuals from MIMIC-IV and a general-ward cohort from our hospital—we demonstrate that the RDW/Alb ratio is an independent and potent predictor of 365-day all-cause mortality. The association remained robust after multivariable adjustment and was successfully validated in an external, non-ICU population, supporting the generalizability and clinical utility of this composite biomarker. By contrast, the TyG index showed no prognostic discrimination in this critically ill population, underscoring RDW/Alb as a low-cost, bedside biomarker that refines risk stratification beyond traditional metabolic indicators.

Our findings indicate that an elevated RDW/Albumin (RDW/Alb) ratio is a robust predictor of poor outcomes in HFpEF. Mechanistically, this ratio reflects three intertwined biological pathways—inflammation, oxidative stress, and malnutrition/hepatic dysfunction—which are increasingly recognized as key drivers of HFpEF progression (17, 18).

**Inflammation mechanism**—studies have shown that circulating levels of interleukin-6 (IL-6), tumor necrosis factor-alpha (TNF-α), and other cytokines are significantly elevated in these patients and correlate with worse haemodynamics and reduced exercise tolerance. IL-6 and TNF-α stimulate hepatic hepcidin production and directly inhibit erythroid progenitor maturation, leading to ineffective erythropoiesis and anisocytosis; the resulting heterogeneity in red blood cell size manifests clinically as an elevated RDW (19–25). These cytokines also suppress hepatic albumin synthesis, and albumin—acting as a negative acute-phase reactant—declines during sustained inflammatory and oxidative stress (26, 27). As a result, inflammation simultaneously increases RDW and lowers albumin, embedding two complementary pathophysiologic signals—erythropoietic stress and impaired nutritional/inflammatory status—into a single composite marker. Given that these disturbances are particularly pronounced in HFpEF, especially among critically ill patients, the RDW/Alb ratio serves as a sensitive reflection of the syndrome's underlying biology and explains its strong, independent association with adverse outcomes in the ICU setting.

**Oxidative stress**—a well-recognized contributor to HFpEF pathophysiology—is driven by comorbidities such as hypertension, type 2 diabetes, and obesity (28–36). These chronic conditions promote mitochondrial dysfunction, excessive production of reactive oxygen species (ROS), and systemic inflammation, collectively disrupting cellular homeostasis in cardiovascular tissues. In erythroid cells, ROS-induced membrane damage and lipid peroxidation compromise red blood cell integrity and deformability, resulting in increased anisocytosis and elevated RDW. Concurrently, oxidative stress accelerates the degradation of serum albumin by oxidizing its thiol-containing Cys34 residue, thereby impairing its antioxidant and transport functions and shortening its half-life. This dual effect—raising RDW while reducing albumin—is sensitively captured by the RDW/Alb ratio. Because oxidative stress is an early and central feature of HFpEF (37, 38), particularly in critically ill patients with multiple comorbidities, an elevated RDW/Alb ratio may reflect not only chronic oxidative and inflammatory burden but also predict mortality in this high-risk population.

**Malnutrition/hepatic dysfunction**—recent large-scale evidence underscores the critical role of nutritional status in shaping outcomes across the full spectrum of heart failure phenotypes, including HFpEF. In a real-world cohort of nearly 12,000 heart failure patients—of whom more than 6,900 had HFpEF—malnutrition, defined by a prognostic nutritional index (PNI) < 45, was independently associated with a 1.8- to 2.5-fold increase in mortality risk, irrespective of body mass index (BMI) (39). Notably, while higher BMI exhibited a protective effect consistent with the well-described “obesity paradox,” this benefit was nullified in patients with poor nutritional status: obese individuals with malnutrition had worse outcomes than normal-weight patients with preserved nutrition (40). PNI, which incorporates serum albumin and lymphocyte count, emerged as a strong and independent predictor of survival, further highlighting the prognostic significance of albumin as a marker of both metabolic reserve and systemic inflammation. These findings offer indirect but compelling support for the biological plausibility of RDW/Alb as a prognostic indicator. A decline in serum albumin reflects not only malnutrition and hepatic congestion but also inflammation-driven suppression of hepatic protein synthesis. At the same time, systemic inflammation and oxidative stress impair erythropoiesis, leading to elevated RDW. The RDW/Alb ratio thus integrates two interrelated prognostic pathways—erythropoietic stress and protein–energy imbalance—into a single, readily available bedside metric (41, 42).

Moreover, endothelial glycocalyx disruption and microvascular dysfunction play a pivotal role in HFpEF pathogenesis. Albumin binds sphingosine-1-phosphate and helps stabilize the endothelial glycocalyx, thereby preserving capillary barrier integrity and nitric oxide signaling. Loss of albumin exacerbates capillary leakage and vascular stiffness (43).

Meanwhile, RDW has been shown to correlate with impaired microvascular perfusion and altered shear stress. The combination of hypoalbuminemia and elevated RDW may therefore reflect underlying microvascular injury. Glycocalyx shedding and coronary microvascular rarefaction are central features in HFpEF, and experimental studies suggest that albumin supplementation can preserve glycocalyx structure and function (44).

Collectively, an elevated RDW/Alb ratio represents a composite signal of (i) chronic inflammation, (ii) oxidative and metabolic stress, and (iii) nutritional-hepatic reserve—three interconnected axes now recognized as primary drivers, rather than mere epiphenomena, of HFpEF progression.

### Limitations of TyG and comparative advantages of RDW/Alb

Although the triglyceride–glucose (TyG) index has shown consistent associations with adverse outcomes in ambulatory and general ward-based HFpEF populations (5, 45), its prognostic value appears to be limited in critically ill HFpEF patients.

Several factors likely account for this discrepancy. First, TyG was originally developed as a surrogate marker of insulin resistance based on fasting triglyceride and glucose levels. In the ICU setting, however, true fasting samples are rarely obtainable due to continuous enteral or parenteral nutrition, intravenous dextrose administration, and stress-related hormonal perturbations. These factors introduce substantial intra-individual variability, diminishing the reliability of TyG as a stable metabolic indicator. Second, glycaemic control in critical care is often protocolized: insulin infusions are used to maintain blood glucose within a narrow range (typically 140–180 mg/dL), thereby reducing between-patient variation in glucose values—a key component of the TyG formula (46). Third, triglyceride levels are frequently confounded by ICU-specific factors, such as lipid-based sedatives (e.g., propofol), intravenous nutritional emulsions, or are suppressed in the setting of systemic inflammation and hepatic dysfunction (47). Moreover, triglycerides are not routinely measured in ICU HFpEF patients, further limiting the clinical utility and generalizability of TyG in this context.

In contrast, RDW and albumin are both universally available as part of the standard first-hour laboratory panel in most ICUs worldwide. They incur no additional cost and are less susceptible to short-term fluctuations caused by acute interventions such as insulin infusions or transfusions.

Other commonly proposed indices—such as NLR and PLR—also originate from routine blood counts, but tend to vary substantially in response to fluid shifts, steroid use, infection, or stress, reducing their reproducibility in critical illness.

Finally, the dominant physiological processes driving mortality in ICU-HFpEF patients are less attributable to chronic insulin resistance and more closely linked to systemic inflammation, malnutrition, and multiorgan dysfunction (48). These pathophysiologic domains are more directly captured by the RDW/Alb ratio, which simultaneously reflects erythropoietic stress and impaired nutritional reserve.

Taken together, the analytic stability, universal availability, and pathobiological relevance of RDW/Alb likely account for its clear and graded association with 30-, 90-, and 365-day mortality in our study cohort—whereas TyG failed to retain significance after multivariable adjustment. These advantages support the use of RDW/Alb as a simple, bedside-accessible tool for early risk stratification in critically ill HFpEF patients.

### Comparison with prior literature and unique contributions of the present study

Previous investigations have shown that RDW/Alb robustly predicts adverse outcomes across a variety of cardiovascular and metabolic conditions—including coronary-care-unit patients and diabetes-related complications (49, 50). More recently, it has also been studied in unselected ICU heart failure cohorts derived from MIMIC-IV (16, 51).

However, until now, no study has evaluated RDW/Alb specifically in ICU patients with heart failure with preserved ejection fraction (HFpEF)—a subgroup characterized by older age, heavy comorbidity burden, and distinct pathophysiological features. Importantly, HFpEF differs fundamentally from HFrEF in terms of disease mechanism, prognosis, and therapeutic strategy (52).

Our study fills this critical gap and contributes to the existing literature in three key ways. First, this is the first study to validate the prognostic value of RDW/Alb specifically in ICU patients with HFpEF. In a cohort of over 3,400 critically ill HFpEF patients, we observed a robust, graded association between RDW/Alb and 30-, 90-, and 365-day all-cause mortality, which remained significant after comprehensive multivariable adjustment. This finding confirms the utility of RDW/Alb beyond general and ward-based settings and supports its potential clinical application in high-acuity environments. Second, our study is also the first to directly compare RDW/Alb with the TyG index in this population. While previous TyG studies focused on low-risk HFpEF (53), TyG lost prognostic significance in our ICU-HFpEF group. Although this subgroup analysis involved a relatively small TyG sample, it reflects a key clinical reality: triglyceride levels are often not measured in this population, limiting the generalizability of TyG in the ICU context. In contrast, RDW/Alb remained independently predictive, underscoring its resilience to metabolic fluctuations and measurement limitations under critical illness. Third, by leveraging the high-resolution, time-stamped laboratory data from the MIMIC-IV database—and applying rigorous outcome adjudication—we constructed one of the largest and most granular real-world cohorts for this research question. This approach enhances both the statistical power and external validity of our findings. Our pre-specified subgroup analyses further refine these insights. The association between RDW/Alb and mortality was consistent across clinical subgroups, but particularly strong in females, non-diabetic patients, those not receiving statins, and individuals without atrial fibrillation or chronic kidney disease, with statistically significant interactions at the 30- and 90-day endpoints. Comorbidities such as COPD and sepsis were unevenly represented across RDW/Alb strata in our cohort. These conditions are well known to worsen outcomes in patients with or at risk for heart failure. COPD is characterized by chronic airway inflammation and impaired cardiopulmonary reserve and is strongly associated with higher risks of acute heart failure events and mortality (54). Similarly, patients with HFpEF who develop sepsis demonstrate higher early deterioration, greater need for mechanical ventilation, and increased emergency-department mortality compared with septic patients without HFpEF (55). These observations underscore the clinical importance of simple, integrative markers in multimorbid HFpEF populations, where inflammatory and metabolic stressors accumulate across multiple pathways.Within this context, our study also revealed an age-related crossover pattern: RDW/Alb provided stronger early discrimination in patients younger than 65 years, whereas its prognostic value became more pronounced at 365 days in older patients. This divergence suggests that the inflammatory and nutritional stress captured by RDW/Alb may manifest differently across clinical phenotypes and time horizons. Younger patients may be more susceptible to acute metabolic derangements, whereas older patients may accumulate chronic physiological vulnerability that becomes increasingly relevant during long-term follow-up. Additionally, although ICU admission is a dynamic physiological period, several biological properties of RDW and albumin support the validity of using first-day measurements. RDW reflects the size distribution of erythrocytes with a lifespan of approximately 3–4 months; therefore, its underlying distribution cannot change substantially in response to short-term hemodynamic fluctuations. Longitudinal data further show that RDW elevations after acute illness persist for weeks to months, reinforcing that it is a slow-moving biomarker rather than an immediate responder to resuscitation (56). Albumin may be influenced by acute inflammation and fluid shifts, but meaningful declines typically develop over 24–48 hours rather than within the first few hours of ICU care (57). Accordingly, most contemporary critical-care cohort studies—including MIMIC-based analyses—use admission or first-day laboratory values as the baseline exposure. This practice aligns with established ICU prognostic methodology and is unlikely to introduce systematic bias.

Collectively, these findings advance current understanding by: (i) confirming the applicability of RDW/Alb in a previously unstudied, high-risk population; (ii) clarifying the limitations of TyG in ICU-HFpEF settings; and (iii) supporting RDW/Alb as a pragmatic and accessible tool for early risk stratification in critically ill HFpEF patients.

### Interpretation of RDW/Alb across acute and non-acute settings

Although ICU admission often follows an apparent acute decompensation, HFpEF deterioration rarely occurs abruptly. Most patients experience several days of worsening congestion, systemic inflammation, impaired nutritional intake, or organ dysfunction before reaching the threshold for critical care. Because RDW and albumin are slow-moving biomarkers—reflecting cumulative erythropoietic stress, chronic inflammation, and nutritional reserve—the RDW/Alb ratio at ICU admission represents the integrated biological burden accumulated over this subacute trajectory rather than an immediate response to hemodynamic instability. Albumin typically declines further during acute-on-chronic deterioration, whereas RDW changes more gradually due to the months-long lifespan of erythrocytes, resulting in higher RDW/Alb values in populations presenting with greater physiological compromise. Accordingly, the higher tertile cut-offs observed in the ICU cohort reflect differences in baseline acuity rather than inconsistency of the biomarker.

Despite these differences in absolute values, the prognostic gradient associated with RDW/Alb remained remarkably consistent across settings. To further reinforce this point, we externally validated our findings in a general-ward cohort of 429 non-ICU HFpEF patients. This validation cohort reproduced the stepwise increase in 365-day mortality across RDW/Alb tertiles, with adjusted hazard ratios confirming the independent predictive value of the index. The concordant risk stratification observed in both ICU and non-ICU populations indicates that RDW/Alb does not function merely as a marker of acute physiological stress but rather captures a broader vulnerability phenotype that remains relevant across the spectrum of HFpEF severity. Together, these results underscore the generalizability and clinical applicability of RDW/Alb beyond critical care settings.

### Interpretation of subgroup results

Our subgroup analysis showed that the prognostic association of RDW/albumin appeared stronger in patients without diabetes and in those not receiving statins. A plausible explanation is that, in non-diabetic individuals, RDW and albumin may more directly reflect subacute inflammatory and nutritional stress. In contrast, patients with diabetes commonly exhibit chronic low-grade inflammation, oxidative stress, disordered erythropoiesis, and altered protein metabolism (58), leading to persistently elevated RDW and reduced albumin. These long-standing abnormalities may compress the dynamic range of RDW/Alb and attenuate its discriminatory capacity. Similarly, statin therapy exerts well-recognized pleiotropic effects beyond lipid lowering—including improvement of endothelial function and attenuation of systemic inflammation and oxidative stress—which may partially buffer the biological disturbances captured by RDW/Alb (59). As a result, the risk gradients associated with RDW/Alb become less pronounced among statin users and more evident in those not taking statins. These interpretations align with the observed interaction patterns but should be viewed as exploratory and hypothesis-generating.

## Limitations

First, although MIMIC-IV provides rich, time-stamped clinical data, it originates from a single academic center, which may limit generalizability to other healthcare systems or resource-constrained ICUs. Second, the retrospective design precludes causal inference and remains susceptible to residual confounding, despite extensive multivariable and subgroup adjustments. Third, RDW and albumin levels were assessed only at admission, preventing evaluation of temporal trends or acute fluctuations that may enhance risk prediction. Fourth, several important modifiers—such as nutritional interventions, transfusion practices, and inflammatory cytokine profiles—were incompletely captured, which may have attenuated or exaggerated the observed associations. Fifth, although the inclusion of an external validation cohort enhances generalizability, it was derived from a single-center retrospective dataset with a relatively small sample size and lacked certain ICU-specific covariates such as illness severity scores. Sixth, identifying HFpEF in administrative or database studies is inherently challenging, as ICD coding alone often undercaptures the condition. To mitigate this issue, we used a hybrid strategy that combined ICD codes with extraction of LVEF ≥50% from narrative echocardiography reports. Although some degree of misclassification cannot be entirely excluded, the direction of such bias is expected to be nondifferential with respect to mortality and would therefore tend to attenuate the true association rather than produce spurious findings. Accordingly, the prognostic association observed in our analysis is likely conservative.

These limitations highlight several priorities for future research. Prospective, multicenter studies are needed to validate the prognostic value of RDW/Alb across diverse ICU-HFpEF populations and to determine population-specific thresholds. Incorporating serial measurements may help assess whether dynamic changes in RDW/Alb offer incremental prognostic value beyond single time-point assessments. Additionally, embedding RDW/Alb into machine-learning frameworks—alongside high-frequency physiologic signals and imaging—could yield real-time risk prediction tools with greater precision than conventional scores.

Finally, it is noteworthy that empagliflozin has demonstrated clinical benefit in patients with HFpEF, though its mechanistic pathways remain incompletely understood. Findings from the EMPEROR-Preserved trial indicate that empagliflozin treatment significantly increased serum albumin levels, even in the absence of notable changes in standard liver function tests (60). Additionally, other studies have demonstrated that empagliflozin improves endothelial and cardiomyocyte function in HFpEF, primarily by reducing pro-inflammatory and oxidative pathways as well as protein kinase Gα oxidation (37). These observations are consistent with our study and underscore the prognostic importance of albumin-based markers such as RDW/Alb. Moreover, previous studies have demonstrated that increasing protein intake and addressing malnutrition through dietary interventions may improve outcomes in HFpEF patients (61). Collectively, these insights provide a compelling rationale for future interventional trials aimed at modifying RDW/Alb—through nutritional support and anti-inflammatory strategies—to determine whether improving this ratio can lead to better clinical outcomes in critically ill HFpEF patients.

## Conclusions

In summary, our findings establish RDW/Alb as a readily available, cost-effective, and prognostically informative biomarker for critically ill patients with HFpEF. By integrating signals of inflammation, oxidative stress, and nutritional reserve, this composite index captures key pathophysiologic processes driving adverse outcomes in this high-risk population. Future prospective studies are warranted to validate its utility across broader settings, including both ICU and non-ICU HFpEF populations, and to explore whether interventions targeting the components of RDW/Alb—such as nutritional support or anti-inflammatory therapy—can improve outcomes.

## Data Availability

The raw data supporting the conclusions of this article will be made available by the authors, without undue reservation.
