# Supplementary material for: Association between RDW-to-albumin ratio and mortality in HFpEF: a retrospective study based on MIMIC-IV and external validation
Source: Front Nutr. 2026 Jan 16;12:1653136. doi: 10.3389/fnut.2025.1653136 (PMC12855092; doi:10.3389/fnut.2025.1653136)

**Supplementary materials**

**Supplementary Table 1.** The meaning of ICD-9 and ICD-10 codes for diseases

**Supplementary Table 2.** The variance inflation factor (VIF) of variables

**Supplementary Table 3.** Baseline characteristics of participants stratified by 30-day mortality

**Supplementary Figure 1.** Multivariable Cox regression analysis of RDW/Alb tertiles and 365-day mortality in the external validation cohort

**Supplementary Figure 2.** Subgroup Analysis of RDW/Albumin Ratio (Continuous) for Predicting 1-Year Mortality in the External Validation Cohort

**Supplementary Table 1. The meaning of ICD-9 and ICD-10 codes for diseases**

1. ICD codes used to define HFpEF (heart failure with preserved ejection fraction):

ICD-9-CM:

42830, 42831, 42832, 42833

ICD-10-CM:

I5030, I5031, I5032, I5033

These codes correspond to diastolic heart failure (unspecified, acute, chronic, acute on chronic).

2. ICD codes used to define other heart failure patients (potential HFpEF candidates):

ICD-9-CM:

39891, 40201, 40211, 40291,

40401, 40403, 40411, 40413, 40491, 40493,

4280, 4281, 4289, 42800, 42801, 42802, 42803,

42810, 42811, 42813, 42820, 42821, 42823,

42840, 42841, 42842, 42843

ICD-10-CM:

I110, I130, I132,

I500, I501, I508, I509

These codes include hypertensive heart and renal disease with heart failure, systolic heart failure, unspecified heart failure, and combined systolic/diastolic forms.

To refine the potential HFpEF cohort, we further extracted patients with an ejection fraction (EF) between 50% and 80% from discharge summaries using regular expression matching.

**Supplementary Table 2. The variance inflation factor (VIF) of variables**

| Variable | VIF |
| --- | --- |
| RDW/Albumin | 1.334 |
| Age, years | 1.448 |
| Female | 1.212 |
| Race | 1.08 |
| Weight, kg | 1.363 |
| Diabetes | 1.677 |
| Hypertension | 1.417 |
| Myocardial infarction | 1.149 |
| Coronary artery disease | 1.319 |
| Cancer | 1.119 |
| Atrial fibrillation | 1.313 |
| Chronic kidney disease | 1.712 |
| COPD | 1.104 |
| Sepsis | 1.213 |
| Hyperlipidemia | 1.199 |
| Cerebrovascular disease | 1.049 |
| RRT use | 1.207 |
| Vasoactive drug | 1.457 |
| Ventilator use | 1.494 |
| Heart rate, bpm | 1.116 |
| MAP, mmHg | 1.032 |
| Smoking | 1.132 |
| WBC, 10^9^/L | 1.079 |
| Hemoglobin, g/L | 1.191 |
| Creatinine | 1.608 |
| Potassium | 1.179 |
| Sodium | 1.11 |
| Used digitalis | 1.081 |
| Used diuretic | 1.229 |
| Used amiodarone | 1.142 |
| Used insulin | 1.607 |
| Used statin | 1.343 |
| Used metformin | 1.035 |
| Used MRA | 1.065 |

Abbreviations: MAP, mean arterial pressure; WBC, white blood cell count; MRA, mineralocorticoid receptor antagonist; COPD, chronic obstructive pulmonary disease; RRT, renal replacement therapy

**Supplementary Table 3.** Baseline characteristics of participants stratified by 30-day mortality

| Variable | Overall | Alive | Dead | P |
| --- | --- | --- | --- | --- |
| Heart rate | 84.0 [25.0] | 83.0 [24.0] | 89.0 [29.0] | <0.001 |
| MAP | 76.0 [21.0] | 77.0 [20.0] | 74.0 [24.0] | <0.001 |
| RR | 19.0 [8.0] | 18.0 [8.0] | 20.0 [8.0] | <0.001 |
| SpO2 | 98.0 [5.0] | 98.0 [5.0] | 97.0 [6.0] | <0.001 |
| Age | 74.0 [18.0] | 73.0 [19.0] | 78.0 [15.0] | <0.001 |
| Bicarbonate | 24.0 [7.0] | 25.0 [6.0] | 23.0 [7.0] | <0.001 |
| Chloride | 101.0 [7.0] | 102.0 [7.0] | 101.0 [8.0] | 0.340 |
| Creatinine | 1.2 [1.0] | 1.2 [0.9] | 1.4 [1.4] | <0.001 |
| Glucose | 123.0 [61.0] | 124.0 [59.0] | 121.0 [69.5] | 0.361 |
| Hemoglobin | 10.7 [3.1] | 10.9 [3.0] | 9.9 [3.0] | <0.001 |
| Magnesium | 2.0 [0.4] | 2.0 [0.4] | 2.0 [0.5] | <0.001 |
| Platelet Count | 201.0 [115.0] | 205.0 [112.0] | 186.0 [129.5] | <0.001 |
| Potassium | 4.2 [0.8] | 4.2 [0.8] | 4.3 [1.0] | 0.006 |
| Sodium | 139.0 [5.0] | 139.0 [5.0] | 138.0 [7.0] | 0.502 |
| Urea Nitrogen | 28.0 [26.0] | 26.0 [22.0] | 36.0 [32.0] | <0.001 |
| WBC | 9.3 [6.6] | 9.1 [6.1] | 10.9 [8.8] | <0.001 |
| ALT | 22.0 [24.0] | 22.0 [22.0] | 23.0 [33.0] | 0.141 |
| AST | 29.0 [31.0] | 28.0 [27.0] | 33.0 [51.0] | <0.001 |
| Bilirubin Total | 0.6 [0.6] | 0.6 [0.5] | 0.6 [0.9] | <0.001 |
| Weight | 80.0 [31.2] | 80.5 [31.2] | 77.7 [31.9] | <0.001 |
| Height | 168.0 [15.0] | 168.0 [15.0] | 165.0 [16.0] | <0.001 |
| BMI | 28.7 [10.5] | 28.8 [10.6] | 28.4 [10.3] | 0.070 |
| SOFA score | 7.0 [8.0] | 6.0 [9.0] | 9.0 [9.5] | <0.001 |
| SAPS II score | 63.0 [26.0] | 61.0 [27.0] | 72.0 [26.5] | <0.001 |
| Length of ICU stay, day | 2.5 [3.6] | 2.4 [3.1] | 3.2 [5.5] | <0.001 |
| Length of hospital stay, day | 10.4 [10.4] | 10.6 [10.2] | 9.8 [11.3] | <0.001 |
| Ventilation-free days at 28 days, day | 25.5 [3.6] | 25.6 [3.1] | 24.8 [5.5] | <0.001 |
| Vasopressor-free days at 28 days, day | 25.5 [3.6] | 25.6 [3.1] | 24.8 [5.5] | <0.001 |
| ICU-free days at 28 days, day | 25.5 [3.6] | 25.6 [3.1] | 24.8 [5.5] | <0.001 |
| OASIS total | 38.0 [10.0] | 37.0 [9.0] | 43.0 [12.0] | <0.001 |
| RDW | 15.0 [2.8] | 14.8 [2.5] | 15.9 [3.3] | <0.001 |
| Albumin | 3.4 [0.9] | 3.5 [0.8] | 3.0 [0.9] | <0.001 |
| RRT | 159 (4.6%) | 77 (2.8%) | 82 (12.4%) | <0.001 |
| Vasoactive drug | 1490 (43.4%) | 1141 (41.1%) | 349 (53.0%) | <0.001 |
| Ventilator use | 1779 (51.8%) | 1393 (50.2%) | 386 (58.6%) | <0.001 |
| Smoking | 660 (19.2%) | 562 (20.2%) | 98 (14.9%) | 0.002 |
| Gender | 1672 (48.7%) | 1360 (49.0%) | 312 (47.3%) | 0.478 |
| Race | 2421 (70.5%) | 1974 (71.1%) | 447 (67.8%) | 0.110 |
| Used aspirin | 2270 (66.1%) | 1942 (69.9%) | 328 (49.8%) | <0.001 |
| Used clopidogrel | 622 (18.1%) | 539 (19.4%) | 83 (12.6%) | <0.001 |
| Used dabigatran | 26 (0.8%) | 22 (0.8%) | 4 (0.6%) | 0.804 |
| Used rivaroxaban | 99 (2.9%) | 88 (3.2%) | 11 (1.7%) | 0.052 |
| Used warfarin | 1023 (29.8%) | 924 (33.3%) | 99 (15.0%) | <0.001 |
| Used heparin | 3172 (92.3%) | 2574 (92.7%) | 598 (90.7%) | 0.108 |
| Used beta blocker | 2689 (78.3%) | 2265 (81.6%) | 424 (64.3%) | <0.001 |
| Used ACEI or ARB | 1254 (36.5%) | 1144 (41.2%) | 110 (16.7%) | <0.001 |
| Used ARNI | 13 (0.4%) | 11 (0.4%) | 2 (0.3%) | 1.000 |
| Used ccb | 1328 (38.6%) | 1138 (41.0%) | 190 (28.8%) | <0.001 |
| Used digitalis | 316 (9.2%) | 241 (8.7%) | 75 (11.4%) | 0.037 |
| Used diuretic | 2985 (86.9%) | 2459 (88.5%) | 526 (79.8%) | <0.001 |
| Used amiodarone | 697 (20.3%) | 555 (20.0%) | 142 (21.5%) | 0.399 |
| Used insulin | 2416 (70.3%) | 1995 (71.8%) | 421 (63.9%) | <0.001 |
| Used statin | 2190 (63.7%) | 1826 (65.8%) | 364 (55.2%) | <0.001 |
| Used sglt2i | 7 (0.2%) | 7 (0.3%) | 0 (0.0%) | 0.359 |
| Used metformin | 140 (4.1%) | 138 (5.0%) | 2 (0.3%) | <0.001 |
| Used MRA | 307 (8.9%) | 265 (9.5%) | 42 (6.4%) | 0.013 |
| Diabetes | 1455 (42.3%) | 1188 (42.8%) | 267 (40.5%) | 0.311 |
| Hypertension | 819 (23.8%) | 701 (25.2%) | 118 (17.9%) | <0.001 |
| Myocardial infarction | 588 (17.1%) | 457 (16.5%) | 131 (19.9%) | 0.041 |
| Coronary artery disease | 1493 (43.5%) | 1243 (44.8%) | 250 (37.9%) | 0.002 |
| Cancer | 507 (14.8%) | 343 (12.4%) | 164 (24.9%) | <0.001 |
| Atrial fibrillation | 1040 (30.3%) | 798 (28.7%) | 242 (36.7%) | <0.001 |
| Acute kidney injury | 1761 (51.3%) | 1314 (47.3%) | 447 (67.8%) | <0.001 |
| Chronic kidney disease | 1429 (41.6%) | 1115 (40.2%) | 314 (47.6%) | <0.001 |
| COPD | 479 (13.9%) | 358 (12.9%) | 121 (18.4%) | <0.001 |
| Sepsis | 445 (13.0%) | 268 (9.7%) | 177 (26.9%) | <0.001 |
| Hyperlipidemia | 1807 (52.6%) | 1496 (53.9%) | 311 (47.2%) | 0.002 |
| Hyperthyroidism | 32 (0.9%) | 24 (0.9%) | 8 (1.2%) | 0.539 |
| Cerebrovascular disease | 234 (6.8%) | 178 (6.4%) | 56 (8.5%) | 0.068 |
| Paraplegia | 22 (0.6%) | 19 (0.7%) | 3 (0.5%) | 0.785 |

Continuous variables are presented as mean ± standard deviation or median (interquartile range), as appropriate based on the distribution of each variable; categorical variables are shown as number (%).

Abbreviations: BMI, body mass index; MAP, mean arterial pressure; RR, respiratory rate; SpO₂, peripheral capillary oxygen saturation; WBC, white blood cell count; ALT, alanine aminotransferase; AST, aspartate aminotransferase; ACEI, angiotensin-converting enzyme inhibitor; ARB, angiotensin receptor blocker; ARNI, angiotensin receptor–neprilysin inhibitor; CCB, calcium channel blocker; SGLT2-i, sodium–glucose cotransporter-2 inhibitor; MRA, mineralocorticoid receptor antagonist; COPD, chronic obstructive pulmonary disease; ICU, intensive care unit; SOFA, Sequential Organ Failure Assessment; SAPS II, Simplified Acute Physiology Score II; OASIS, Oxford Acute Severity of Illness Score; RRT, renal replacement therapy; LOS, length of stay.

**Supplementary Figure 1.** Multivariable Cox regression analysis of RDW/Alb tertiles and 365-day mortality in the external validation cohort


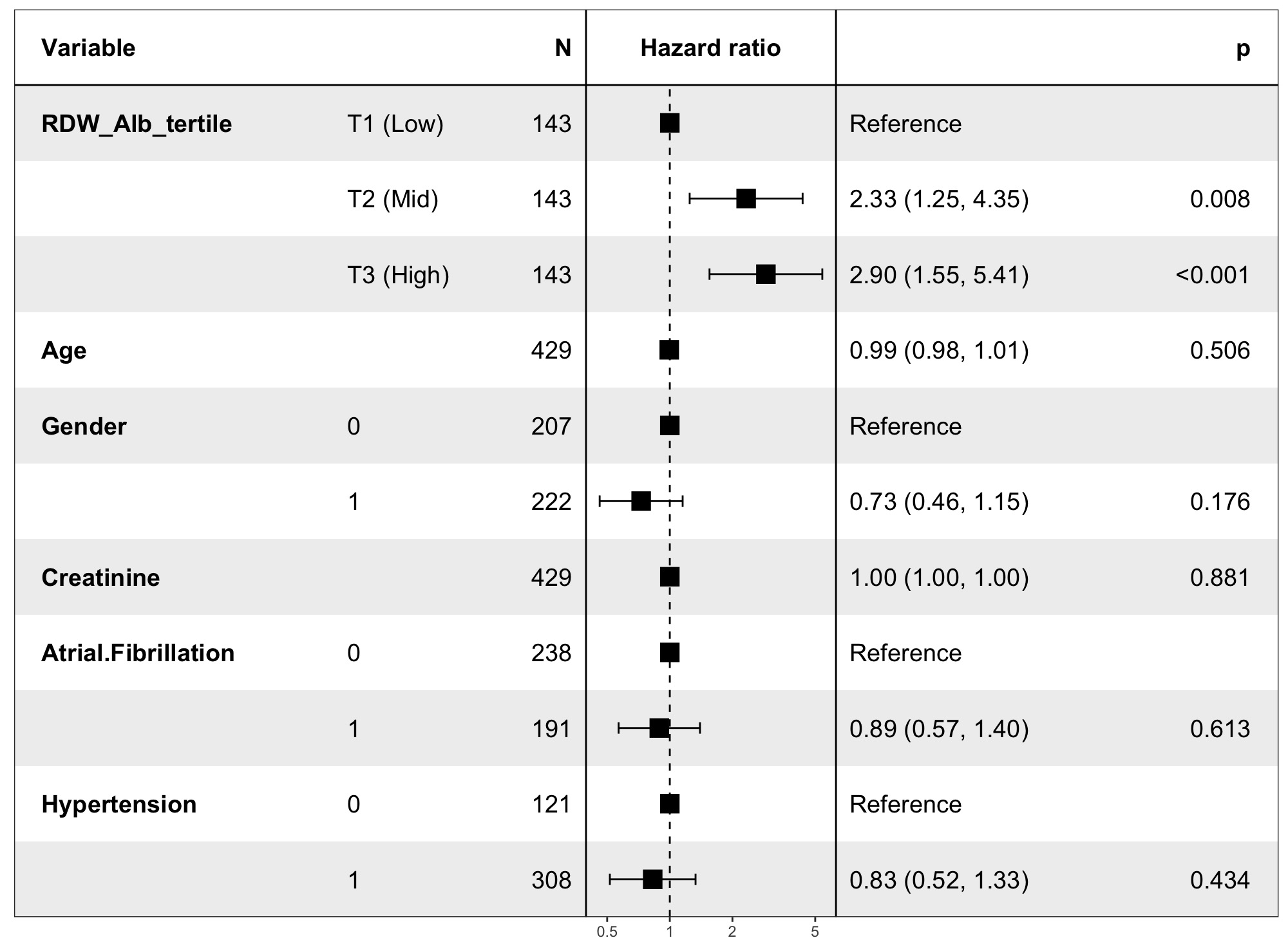


**Supplementary Figure 2.** Subgroup Analysis of RDW/Albumin Ratio (Continuous) for Predicting 1-Year Mortality in the External Validation Cohort


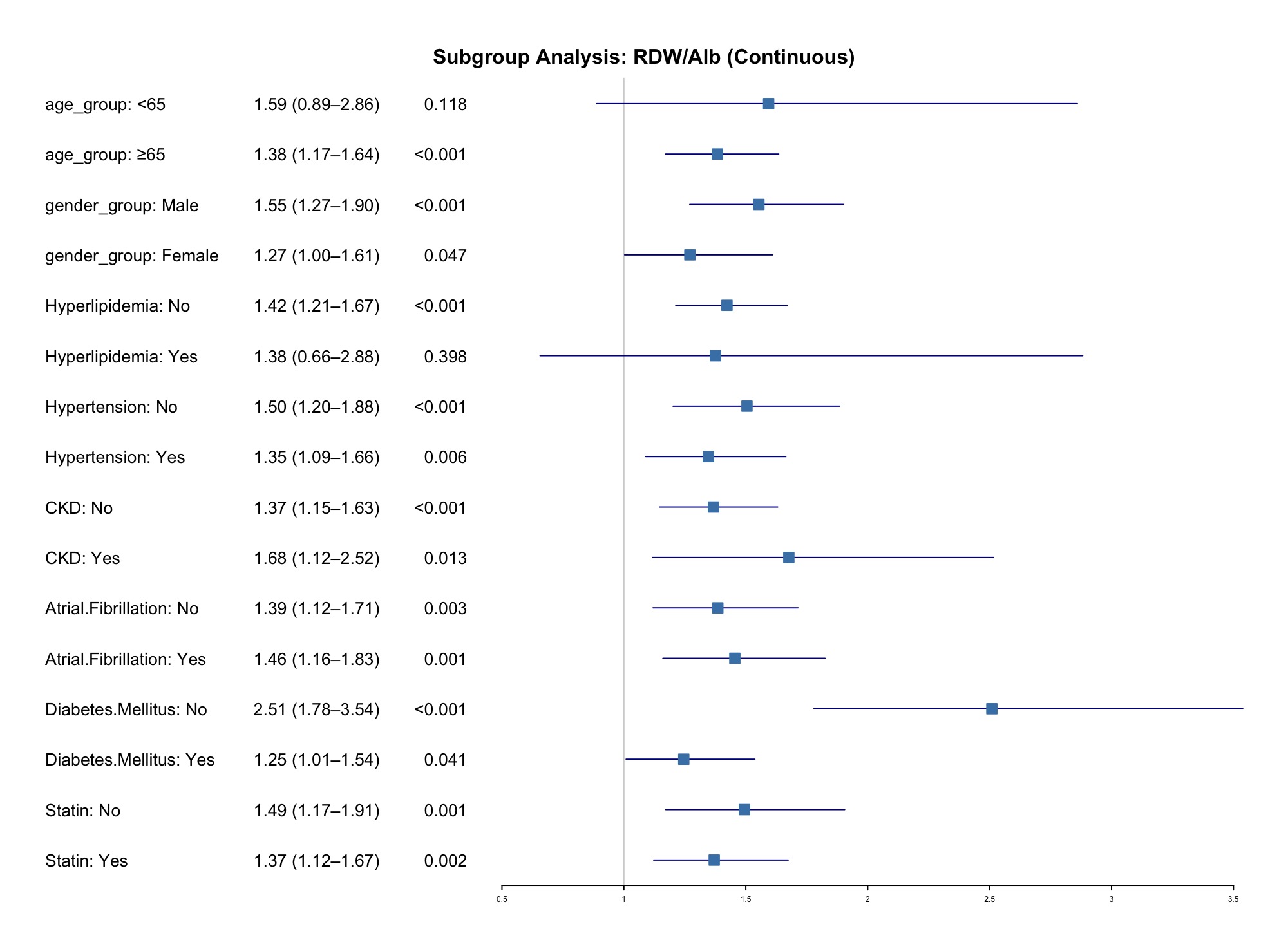

Supplement: Supplementary file 1 [file Table_1.docx]
